# Are microRNAs Important Players in HIV-1 Infection? An Update

**DOI:** 10.3390/v10030110

**Published:** 2018-03-03

**Authors:** Muthukumar Balasubramaniam, Jui Pandhare, Chandravanu Dash

**Affiliations:** 1 Center for AIDS Health Disparities Research, Tennessee Center for AIDS Research; Meharry Medical College, Nashville, TN 37208, USA; muthukumarb@mmc.edu (M.B.); jpandhare@mmc.edu (J.P.); 2 Department of Biochemistry and Cancer Biology, Meharry Medical College, Nashville, TN 37208, USA; 3 Department of Graduate Studies and Research, Meharry Medical College, Nashville, TN 37208, USA

**Keywords:** miRNA, HIV, Post-transcriptional regulation

## Abstract

HIV-1 has already claimed over 35 million human lives globally. No curative treatments are currently available, and the only treatment option for over 36 million people currently living with HIV/AIDS are antiretroviral drugs that disrupt the function of virus-encoded proteins. However, such virus-targeted therapeutic strategies are constrained by the ability of the virus to develop drug-resistance. Despite major advances in HIV/AIDS research over the years, substantial knowledge gaps exist in many aspects of HIV-1 replication, especially its interaction with the host. Hence, understanding the mechanistic details of virus–host interactions may lead to novel therapeutic strategies for the prevention and/or management of HIV/AIDS. Notably, unprecedented progress in deciphering host gene silencing processes mediated by several classes of cellular small non-coding RNAs (sncRNA) presents a promising and timely opportunity for developing non-traditional antiviral therapeutic strategies. Cellular microRNAs (miRNA) belong to one such important class of sncRNAs that regulate protein synthesis. Evidence is mounting that cellular miRNAs play important roles in viral replication, either usurped by the virus to promote its replication or employed by the host to control viral infection by directly targeting the viral genome or by targeting cellular proteins required for productive virus replication. In this review, we summarize the findings to date on the role of miRNAs in HIV-1 biology.

## 1. Introduction

The human immunodeficiency virus 1 (HIV-1) belongs to the *Lentivirus* subfamily of retroviruses and causes the acquired immunodeficiency syndrome (AIDS). HIV-1 primarily targets and replicates in the cluster of differentiation 4^+^ (CD4^+^) T-lymphocytes and cells of the monocyte/macrophage lineage. HIV-1 pathogenesis progresses through three major stages: acute, chronic, and AIDS [1]. During the acute infection phase that typically lasts one to two months, the virus replicates rapidly and, by triggering multiple cell death pathways, causes loss of the CD4^+^ T-cells [2]. The subsequent chronic infection phase is marked by generalized immune activation and low levels of virus replication, but is accompanied by a sustained loss of CD4^+^ T-cells [3]. It might be several years (10 years on average) before the infection progresses to AIDS, which is characterized by low CD4^+^ T cell counts and the development of opportunistic infections and tumors. 

HIV-1 infections have reached pandemic proportions since an ancestral form of the virus entered the human population over a century ago through zoonotic transmission from a simian immunodeficiency virus (SIV)-infected chimpanzee in central Africa [4,5,6]. The ongoing global HIV pandemic has already claimed over 35 million lives, with Sub-Saharan Africa, home to nearly 70% of HIV-1 infected people, continuing to bear the brunt. While an effective anti-HIV vaccine remains elusive [7,8,9], an estimated 1.8 million people were infected with HIV-1 in 2016 and millions continue to die each year (one million deaths in 2016) from HIV/AIDS-related illnesses worldwide. The only recourse for the more than 36 million people currently infected with HIV-1 are antiretroviral drugs (ARV) that inhibit various stages of the virus life cycle [10]. However, drug-resistant HIV-1 strains rapidly emerge due to mutations in the viral genome that largely ensue from a combination of high replication rate and low-fidelity of the viral enzyme that copies the viral RNA genome into a double-stranded complementary DNA (cDNA) and, potentially, due to low-fidelity of the host DNA-dependent RNA polymerase II that transcribes the integrated viral DNA genome [11,12]. Hence, a combination antiretroviral therapy (cART) comprising of a cocktail of potent ARVs is widely used for treatment to minimize the emergence of drug resistance [10]. The resultant reduction in viral load, restoration or maintenance of immunocompetence, prevention of or delay in the onset of AIDS, and improved quality of life, have all together significantly reduced HIV/AIDS-associated mortality and morbidity rates [13]. Nevertheless, persistence of stable viral reservoirs in resting memory CD4^+^ T-cells (HIV-1 latency) has precluded continued suppression of viral load to undetectable levels upon cessation of cART (functional cure) or eradication of the virus (sterilizing cure) [14,15,16,17,18,19,20,21,22,23,24]. The prevalence of drug-resistant HIV-1 strains [25,26,27,28] and the enduring challenge posed by the latent viral reservoirs necessitates urgent development of novel ARVs that target unexploited stages of HIV-1 life cycle [29].

Both viral and host factors determine the clinical course of HIV-1 infection [1,30,31,32,33,34,35,36,37]. Recent progress in identifying and determining the role of host factors and cellular processes in virus biogenesis and transmission underscore the prospect of novel and promising therapeutic strategies for the prevention and/or management of, and possibly cure for, HIV/AIDS [38,39]. Notably, the cellular gene silencing process termed RNA interference (RNAi), which is mediated by small non-coding RNAs (sncRNAs) and can modulate gene expression at the posttranscriptional level, is increasingly being recognized as a prime platform for developing and delivering non-traditional antiviral therapeutic strategies.

In this review, we start with a primer on the HIV-1 replication cycle in target cells, followed by a concise overview of the nature and role of cellular sncRNAs in host gene expression, and an outline of the current understanding of the miRNA biogenesis. We then discuss the reported roles of cellular miRNAs as cofactors or inhibitors of HIV-1 replication, all the while drawing upon, to provide a broader context and the current status of this rapidly evolving field, topical findings from studies on other viruses. We also address the ongoing debate on whether HIV-1 encodes miRNAs and, if so, their functional relevance. We note here that several reviews discussing the interplay between viruses and cellular miRNA machinery have been published in the recent past [40,41,42,43,44,45,46,47,48,49].

## 2. HIV-1 Replication

Three decades of intense research on HIV/AIDS has considerably improved our understanding of virus infection and transmission [23]. Basic research on HIV/AIDS has made inroads into several aspects of the replication strategy employed by the virus [50]. A relatively small virus (~100–120 nm), the spherical HIV-1 particles contain two copies of non-covalently linked positive-sense single-stranded RNA genome of ~10 kilobases (Kb) size [51,52]. This viral genomic RNA (vRNA), harboring several functional secondary structures, contains nine genes encoding 15 canonical proteins [53,54]. As an obligate intracellular parasite, HIV-1 relies on host cell machinery and proteins for its productive replication in infected cells. Remarkably, over 2000 host cellular proteins, termed host-dependency factors (HDFs), have been implicated in HIV-1 replication cycle [55,56]. The virus also encounters, and in most cases, overcomes the restriction imposed by the host-encoded intrinsic and innate immune factors [34,36,57,58]. To gain entry into the target cell, HIV-1 uses the viral membrane-anchored envelope glycoprotein (gp120) to first bind to the CD4 receptor and, then, the C-C chemokine receptor type 5 (CCR5) or C-X-C chemokine receptor type 4 (CXCR4) chemokine co-receptor on the target cell surface [59]. These virus–cell interactions promote fusion of their respective membranes thereby allowing entry and cytoplasmic release of the viral “core”—a cone-shaped protein shell composed of polymer of the viral capsid protein (CA) [60,61,62,63,64]. The viral core harbors the viral RNA genome, virally-encoded enzymes and proteins, and host proteins; the viral enzymes reverse transcriptase (RT) and integrase (IN) are essential for mediating the next steps in viral replication. The viral RT, functioning as a component of the nucleoprotein complex termed reverse transcription complex (RTC), synthesizes a double-stranded cDNA (vDNA) copy of the vRNA [11]. This reverse transcription process is intricately synchronized with the disassembly of the CA shell of the viral core (uncoating), a highly ordered, but poorly understood, process that regulates the subsequent early post-entry steps of viral replication including nuclear entry and integration of the vDNA [62,65,66]. The viral IN, functioning as a component of another nucleoprotein complex termed pre-integration complex (PIC), traffics the vDNA into the nucleus and enables its integration into the host chromosome [67,68,69]. The integrated vDNA (provirus) may then, be transcribed or remain silent as latent provirus [22,70]. The cellular RNA polymerase II transcribes the proviral DNA into viral mRNAs, a population of which undergoes further processing including splicing. The host cell machinery mediates the nuclear export of the differentially spliced viral mRNAs whereas virus-encoded proteins facilitate the transport of the unspliced full-length vRNA to the cytoplasm [71]. The viral mRNAs are translated into the respective viral proteins competent for structural (Gag polyprotein (Pr55Gag), Gag-Pro-Pol polyprotein (Pr160GagPol), envelope glycoprotein polyprotein (gp160)), accessory (Nef, Vif, Vpu, Vpr) and regulatory (Tat, Rev) roles. Subsequently, viral constituents necessary for the generation of progeny virions including the Gag, Gag-Pro-Pol, envelope glycoproteins, and the vRNA traffic to specific intracellular membrane niches, where the Gag orchestrates their assembly into immature virus-like particles (VLPs) [72,73,74,75,76]. The cellular endosomal sorting complexes required for transport (ESCRT) machinery mediates the subsequent budding and pinching of the assembled VLPs into the extracellular milieu and the concomitant PR-mediated maturation process yields infectious progeny virus (virions) [72,77,78,79]. During the assembly and budding process, several host RNAs and proteins from the producer cell are incorporated into the virus particle [80,81,82]. Finally, transmission of the progeny virions leads to the establishment of new infections [83,84].

## 3. Regulation of Cellular Gene Expression by Small Non-Coding RNAs

Gene expression is regulated at transcriptional, post-transcriptional, and post-translational stages. Research in the past two decades has uncovered RNAi, a key and sophisticated cellular pathway in eukaryotes that mediates gene silencing via the action of sequence-specific sncRNAs [85,86]. Three major classes of sncRNAs that mediate RNAi in animals include the small interfering RNAs (siRNAs), microRNAs (miRNAs), and Piwi-interacting RNAs (piRNAs) [87,88,89,90,91]. Additional types of sncRNAs possessing diverse biological roles continue to be discovered. RNAi-mediated gene silencing can be executed at the level of transcription—termed transcriptional gene silencing (TGS), or post-transcription—termed post-transcriptional gene silencing (PTGS). TGS involves stable repression of transcription via DNA methylation or histone modification and has generally been recognized as a cellular gene regulatory mechanism in lower eukaryotes and plants. RNAi-mediated PTGS involves sncRNAs acting in concert with the Argonaute (AGO) family proteins to form the multi-protein RNA-induced silencing complexes (RISCs), which then cleave the transcript or inhibit translation [92].

**Figure 1:**
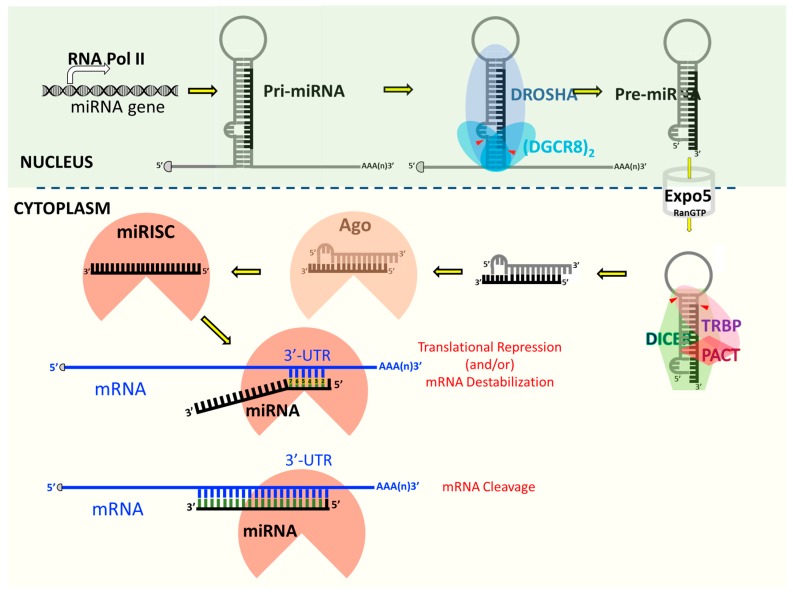
Schematic representation of the canonical microRNA (miRNA) biogenesis pathway in humans: The RNA Polymerase II transcribes the nuclear chromosome-encoded miRNA gene into primary miRNA (pri-miRNA) that comprises a stem, a terminal loop, and single-stranded 5′ and 3′ RNA tails. The nuclear microprocessor complex-resident Drosha, in concert with its cofactor DGCR8, recognizes the pri-miRNA and cleaves asymmetrically at defined sites in the stem. The released stem-loop structure termed precursor miRNA (pre-miRNA) is then transported to the cytoplasm by the Exportin 5-RanGTP protein complex. The cytoplasmic Dicer, in concert with potential cofactors like transactivating response RNA-binding protein (TRBP) and protein activator of the interferon-induced protein kinase (PACT), recognizes the 2-nucleotide 3′ overhang and/or the 5′ phosphorylated end of the pre-miRNA and cleaves at a defined distance from those termini. The ensuing asymmetric ~22-nucleotides-long miRNA duplex, marked by the 5′ monophosphate and a 2-nt 3′ overhang on both strands, is then loaded onto the AGO protein complex in an ATP-dependent process aided by chaperones and co-chaperones. The miRNA duplexes are then unwound and one of the strands (passenger strand), selected on the basis of 5′ nucleotide composition and thermodynamic stability, concomitantly undergoes dissociation or degradation. The resultant mature miRNA (guide strand), in the context of the effector nucleoprotein complex called miRNA-induced silencing complex (miRISC), then binds the target mRNA leading to translational suppression or mRNA deadenylation or mRNA degradation.

The siRNAs and miRNAs are generated by the action of cellular RNase III enzymes and are posttranscriptional regulators of gene expression [93,94,95,96,97]. However, they exhibit structural, physiological and functional differences, and their target mRNAs face different fates [98]. The siRNAs are ~21-nucleotides-long double-stranded (ds) sncRNA molecules derived by sequential exonucleolytic processing of longer, linear, completely complementary dsRNA precursors by the RNAse III enzyme Dicer. One strand of the siRNA, termed as the “guide strand”, then incorporates into and guides the RISC to the target mRNA displaying perfect sequence similarity. The RISC then endonucleolytically cleaves and degrades the target mRNA. This sequence complementarity-dependent activity of siRNAs ensures knockdown of specific genes. The siRNAs derived from endogenous or exogenous dsRNAs have been categorically shown to silence host genes or viral genes in plants and invertebrates, both of which lack any antibody or cell-mediated immunity. However, their existence and function in mammals, especially in somatic cells, remains ill-defined and controversial. In mammalian cells, siRNAs typically arise from exogenous dsRNAs, which typically are viral replication intermediates, imperfect RNA hairpins in the viral genome, or experimentally introduced substrates. Unlike the siRNAs, mature functional miRNAs are derived from longer, incompletely complimentary stemloop dsRNA precursors termed primary miRNA (pri-miRNA), which are either encoded by cellular genes located primarily in the intergenic regions or present in the introns intervening the protein-coding genes. Downstream sequential processing steps involving multiple proteins or protein complexes yield the ~22-nucleotides-long single-stranded mature miRNAs (Figure 1) (for detailed discussion on miRNA biogenesis, please see Section 4). The mature miRNAs harbor a “seed region”, defined by nucleotides 2–8, that recognizes and binds perfectly complementary sequences in the target mRNAs; binding beyond this seed region is generally based on partial complementarity. The bound miRNAs regulate the expression of target mRNAs at the post-transcriptional level via translational repression and/or mRNA deadenylation or decay. The miRNAs exhibit partial complementarity to target mRNAs and hence may regulate hundreds of target genes. Further, miRNAs have been shown to occur and function exclusively in eukaryotes—plants and animals. In contrast to the siRNAs and the miRNAs, the ~24–30 nucleotides-long single-stranded piRNAs are generated from single-stranded RNA precursors independent of any RNase III enzyme activity. The piRNAs are specifically expressed in germ line cells of vertebrates and invertebrates, wherein they bind PIWI subfamily members of the AGO family proteins and the resulting RNA-protein complexes silence, primarily, the transposons [89,99,100].

## 4. Cellular microRNAs

miRNAs modulate diverse cellular processes including development, differentiation, metabolism, proliferation, tumorigenesis, immune response, and cell death [101,102]. Many miRNA genes are conserved from *C. elegans* to humans. To date, the human genome has been reported to encode anywhere from 500 (MirGeneDB, v1.1 [103]) to over 1880 miRNAs (miRBase, Release 21 [104]), randomly distributed in all chromosomes except for the Y chromosome. Interestingly, a recent study of 13 different human tissue samples revealed the presence of 3707 novel miRNAs, of which over half were human-specific [105]. However, the functional relevance of the majority of these miRNA annotations is currently unknown. Nevertheless, miRNAs have been projected to regulate the expression of up to 60% of mammalian [106,107,108] or 90% of human protein-encoding genes [109]. Because miRNAs are only partially complementary to their target mRNAs, each miRNA is estimated to be capable of regulating the expression of up to 200 genes [110,111]. Conversely, binding sites for multiple miRNAs have been reported to be present on individual target mRNAs [112]. In the canonical miRNA-processing pathway, specific transcription factors stimulate the transcription of the primary miRNA (pri-miRNA) transcripts from miRNA-encoding genes located within the introns or 3′-untranslated region (UTR) of coding or non-coding mRNAs, primarily by RNA polymerase II [113,114]. However, RNA polymerase III catalyzes the transcription of pri-miRNAs corresponding to certain miRNAs, including those encoded within Alu repeat sequences and by some viruses. A large percentage of human miRNAs (~40%) are cotranscribed as miRNA clusters, i.e., a single pri-miRNA transcript harbors multiple miRNA sequences (Figure 1). The pri-miRNAs harbor stem loop (hairpin) structures and undergo sequential processing, first in the nucleus and then in the cytoplasm. In the nucleus of mammalian cells, a heterotrimeric “microprocessor” complex, containing one copy of the RNase III endonuclease Drosha and two copies of its cofactor the ds-RNA binding protein DiGeorge syndrome critical region gene 8 (DGCR8), cleaves the pri-miRNA ~11 bp from the basal junction and ~22 bp from the apical junction [115,116,117,118,119,120]. This releases on average ~70-nucleotides-long (may range from ~60–110 nucleotides) hairpin precursors known as precursor miRNAs (pre-miRNAs) that harbor a two-nucleotide 3′ overhang. The pre-miRNAs are then exported by a protein complex containing Exportin 5 and RanGTP into the cytoplasm [121,122,123]. In the cytoplasm of mammalian cells, the RNase-III family enzyme Dicer, in concert with HIV-transactivating response RNA-binding protein 2 (TRBP2) and protein activator of the interferon-induced protein kinase (PACT), recognizes the pre-miRNA’s 2 nucleotide 3′ overhang and cleaves off the loop structure ~22 nucleotides from the Drosha cleavage site [124,125,126,127,128,129,130,131,132]. The released asymmetric ~22-nucleotides-long miRNA duplexes harbor a 5′ monophosphate and a two-nt 3′ overhang on both strands-features that help them get loaded, aided by heat shock cognate 70 kDa (Hsc70)/heat shock protein 90 (Hsp90) chaperones and their co-chaperones, onto the AGO protein complex [133,134,135,136]. The miRNA duplexes are then unwound and, in a strand selection process based on 5′ nucleotide composition and thermodynamic stability, the concomitant dissociation or degradation of one of the strands (passenger strand) finally yields the mature miRNA (guide strand); the ensuing effector nucleoprotein complexes are called the miRNA-induced silencing complexes (miRISC) [137,138,139,140,141,142]. A seed region comprising of nucleotides 2–8 located in the 5′ region of the guide miRNA strand determines its ability to recognize the target mRNA harboring complementary sequences [143,144,145,146,147]. This biologically active mature miRNA guides the miRISC complex to bind the target mRNA, typically in the 3′UTR but potentially in the coding region [148] and in the 5′UTR [149] and cause translational suppression or mRNA deadenylation or mRNA degradation [88,150,151,152,153,154,155,156]. Recent research has also identified miRNA biosynthetic pathways in mammalian cells that deviate from the canonical pathway, thereby adding multiple layers of complexity to the miRNA-mediated gene regulation. Such alternative miRNA biosynthesis pathways may be Drosha/DGCR8-independent (e.g., Mirtrons), Dicer-independent (e.g., miR-451), and both Drosha/DGCR8- and Dicer-independent (e.g., Agotrons) [157,158,159,160,161,162,163,164].

## 5. Cellular miRNAs as Cofactors in HIV-1 Replication

Viruses generally encode minimal genetic information, and hence depend on host cellular factors and pathways to complete productive replication. Studies utilizing genomic, transcriptomic, and proteomic approaches have identified hundreds, and in some studies, thousands of host cell factors involved in different stages of virus replication. Interestingly, striking overlaps exist between the cellular pathways usurped by the virus and those regulated by the miRNAs. Accordingly, cellular miRNAs have been reported to affect virus infections in a positive or negative manner.

Cellular miRNAs can positively impact virus replication directly by associating with the virus or viral components to counter the antiviral pathways. A striking example, and a first report involving an RNA virus, was the demonstration that the liver-specific miR-122 binds directly to the 5′-UTR of the hepatitis C virus (HCV) RNA without affecting the viral mRNA stability or translation. Interestingly, the virus replication was reduced when the miR-122 binding to HCV RNA was blocked, thus indicating that miR-122 promoted HCV replication [165]. Subsequently, miR-122, in concert with AGO2, was shown to protect the bound HCV RNA from host mRNA decay machinery [166,167]. Interestingly, miR-122 was recently shown to primarily enhance the viral RNA synthesis by competing with the poly(rC)-binding protein (PCBP2), thus steering the viral genome away from translation [168]; this, albeit indirectly, reinforces the canonical role of miRNAs as translational repressors. The recognition of miR-122 as an essential cofactor for HCV replication has resulted in the development, and clinical trial, of an miR-122-targeting antimiR. In an example of a miRNA targeting the 3′-UTR of RNA viruses, the bovine viral diarrhea virus (BVDV) and classical swine fever virus (CSFV) were shown to rely on the binding of cellular miR-17 family to the viral 3′-UTR towards promoting viral RNA stability and translation [169]. Cellular miRNAs may also be sequestered by viruses, thereby protecting cellular cofactors from miRNA-mediated downregulation. Interestingly, both miR-122 and miR-17 has been shown to act as “miRNA sponges”, thus altering the host transcriptome [169,170]. Further, the hepatitis B virus (HBV) RNA harbors six miR-122-binding sites that act as sponges of miR-122; the ensuing sequestration and downregulation of miR-122 was proposed to steer them away from their inhibitory action on cellular proteins that aid HBV replication [171,172]. Notably, to date no cellular miRNA has been demonstrated to promote HIV-1 replication by directly associating with the vRNA.

Cellular miRNAs can also positively impact virus replication in an indirect manner, as evidenced by the induction of such miRNAs by some viruses. In an example of an RNA virus employing cellular miRNA to selectively disrupt host machinery, the enterovirus EV71 was shown to upregulate cellular miR-141, which binds the 3′UTR of the eukaryotic translation initiation factor 4E (eIF4E) mRNA and downregulates its expression, thereby facilitating virus-mediated switch from cellular to viral translation [173]. The eastern equine encephalitis virus (EEEV) has reportedly evolved an intriguing replication strategy wherein binding of the cellular miR-142-3p to the 3′UTR of the viral RNA and the ensuing suppression of viral replication was proposed to aid the virus avoid triggering the host interferon (IFN) system [174]. Interestingly, the Epstein-Barr virus (EBV) appears to have multiple options in cellular miRNAs towards establishing and maintaining latent infections. The EBV induces expression of several cellular miRNAs including miR-155 [175] and let-7a [176]; while miR-155 suppresses the expression of cellular proteins mediating cell cycle arrest and apoptosis, thus ensuring EBV latency, the let-7a downregulates Dicer and, as a consequence, its positive effect on EBV reactivation. Conversely, the cellular miRNAs miR-200b and miR-429 were shown to down-regulate the expression of two cellular proteins, zinc finger E-box binding homeobox 1 (ZEB1) and , zinc finger E-box binding homeobox 2 (ZEB2), known to play critical roles in the maintenance of EBV latency, thereby relieving the block on virus reactivation from latency and production of infectious virus [177].

Chiang et al. [178] recently reported the first cellular miRNA that appears to promote HIV-1 replication in a CD4^+^ T-cell line. Activation of CD4^+^ T-cells is a prerequisite for productive HIV-1 infection and entails substantial transcriptional changes, including alterations in cellular miRNA expression. Interestingly, CD4^+^ T-cell activation has been shown to diminish cellular levels of several anti-HIV miRNAs that directly target the viral RNA [179,180] or inhibit cellular cofactors [181,182,183]. So, Chiang et al. [178] hypothesized that miRNAs upregulated upon CD4^+^ T-cell activation might promote HIV-1 replication. Accordingly, when compared to the resting cells, miR-132 levels were significantly upregulated in phytohaemagglutinin (PHA)-activated CD4^+^ T-cells isolated from healthy donors and peripheral blood mononuclear cells (PBMC) from HIV-1 patients. Conversely, overexpression of exogenous miR-132 promoted HIV-1 replication in Jurkat T cells and reactivated HIV-1 in a latently infected Jurkat cells. Interestingly, miR-132 has previously been reported to target and down-regulate a cellular transcriptional regulatory protein called Methyl-CpG-binding protein 2 (MeCP2) [184,185]. In accordance with the miR-132 overexpression data, MeCP2 knockdown augmented HIV-1 production. However, MeCP2 knockdown did not reactivate latent HIV-1, which suggests that miR-132 modulates the biogenesis of HIV-1 and reactivation of latent HIV-1 via two mechanistically different routes. Predating the report by Chiang et al. [178], two cellular miRNAs- miR-34a and miR-217, were reported to bind the 3′UTR, and repress the translation, of the mRNA of silent mating type information regulation 2 homolog 1 (SIRT1) [186,187]. The SIRT1 is a cellular protein deacetylase known to deacetylate the nuclear factor kappa B (NF-kB) and inhibit its positive effect in Tat-induced transactivation of HIV-1 long terminal repeat (LTR). Therefore, the miRNA-induced perturbation of SIRT1′s deacetylation activity resulted in increased acetylation of NF-kB and, consequently, enhanced transactivation of HIV-1 LTR. However, these studies investigated the effect of miRNAs on the transactivation of HIV-1 LTR in the absence of virus infection by using TZM-bl cells (containing integrated HIV-1 LTR-driven Luciferase reporter) or Magi cells (containing integrated HIV-1 LTR-driven beta-galactosidase reporter), or SupT1 cells transfected with LTR-Luc construct.

Additional cellular miRNAs enabling HIV-1 infection by downregulating the levels of host antiviral proteins have been reported. The cyclin-dependent kinase inhibitor protein p21 is known to disrupt HIV-1 gene expression by blocking the transcriptional elongation factor cyclin-dependent kinase 9 (CDK9), and the potassium channel protein TWIK-related acid-sensitive potassium channel 1 (TASK1) impairs the viral protein Vpu-mediated enhancement in HIV-1 release [188,189]. Farberov et al. [190] reported that the downregulation of p21 and TASK1 by the cellular miRNAs Let-7c and miR-124a or miR-34a-5p, respectively, led to increased HIV-1 replication in the JLTRG-R5 (Jurkat-derived cells containing integrated HIV-1 LTR-driven GFP reporter) and HeLa-CCR5 cells. Another member of the cellular miR-34 family, the miR-34c-5p was also recently shown to promote HIV-1 infection in CD4^+^ T-cells [191]. The expression of miR-34c-5p was upregulated during the T cell receptor (TCR) signaling-mediated activation of naïve CD4^+^ T-cells, which altered the expression levels of multiple genes involved in HIV-1 replication. Interestingly, the enhancement of HIV-1 replication in T-cells overexpressing miR-34c-5p and the downregulation of endogenous miR-34c-5p upon HIV-1 infection led the authors to speculate that the latter is an antiviral host response.

## 6. Cellular miRNAs as Anti-Viral Factors in HIV-1 Replication

Cellular miRNAs may also negatively modulate virus replication. This is unsurprising because siRNAs, which inhibit mRNA expression by comparable mechanism, have been shown to effectively impair virus replication. In fungi, plants, and invertebrates, siRNA-mediated RNAi functions as an innate defense response against virus infections. The dsRNA intermediates generated during virus replication, especially that of RNA viruses, are processed by DICER into virus siRNAs (vsiRNAs) that direct the RISC machinery to silence the corresponding viral RNA. Invariably, viruses have evolved to equip themselves with evasion strategies to counter this host-mediated defense response. For instance, viruses are known to generate viral variants that have acquired escape mutations in the RNAi target sequence, and the ensuing sequence noncomplementarity or modified local RNA secondary structure might impair siRNA recognition and binding to the viral target sequence. A more prevalent strategy employed by plant viruses is to encode viral suppressors of RNAi (VSRs) that, besides playing crucial roles in the virus replication, block certain steps of the RNAi pathway via different mechanisms. For example, VSRs might bind to long dsRNA duplexes to block siRNA synthesis or to siRNA duplexes to preclude the RISC formation; they might also directly target the constituents of the RISC machinery or the host enzymes catalyzing DNA methylation.

Though some mammalian viruses have been reported to encode VSR proteins, including influenza A virus (IAV) NS1, Ebola virus (EBOV) VP35, and human enterovirus 71 (HEV71) 3A, the existence of an mammalian RNAi pathway possessing antiviral potential, especially in somatic cells, remains a contentious area of research [192,193,194,195]; the lack of or low levels of vsiRNAs in virus-infected mammalian cells [196,197] and the lack of enhanced virus infection in Dicer-deficient cells [198,199] are often cited in argument against the existence of mammalian antiviral RNAi. Adding to the complexity, reciprocal inhibition between the RNAi machinery and the interferon response pathways involving interferon-stimulated genes (ISGs) has been reported. Seo et al. [200] demonstrated that RNAi represses the expression of ISGs in uninfected cells to avert cytotoxicity, but virus infection and the ensuing antiviral responses relieve the inhibitory effect on the expression of ISGs. This, the authors proposed, indicates that interferons exclusively mediate the cellular response to virus infection. Backes et al. [201] came to similar conclusion after comparing the titers of recombinant viruses capable of disrupting IFN-mediated response or RISC-associated silencing; while impaired IFN response yielded higher virus titers, inactivation of small RNA-mediated silencing in the presence or absence of IFN response lowered the virus titers. These findings led the authors to propose that mammalian response to virus infection is not only independent of RNAi pathway.

However, infection of IFN-nonresponsive mouse embryonic stem cells with encephalomyocarditis virus (EMCV) or Nodamura virus (NoV) [202], and infection of differentiated cells by a NoV mutant lacking its VSR B2, both induced the production of siRNAs [203]. Despite the lack of evidence showing that these siRNAs were functional products of the canonical RNAi machinery and the use of stem cells not naturally targeted by EMCV or NoV, these studies raised the possibility that the prior failed attempts at demonstrating antiviral RNAi in mammals could simplty reflect the masking or inhibitory effect by the IFN pathway and/or by the virus-encoded VSRs. Accordingly, Maillard et al. [204] demonstrated that mammalian somatic cells with impaired IFN pathway were capable of mounting antiviral RNAi, and Li et al. [205] reported that infection by a mutant IAV lacking the VSR NS1, but not by the WT virus, led to generation of vsiRNAs in a Dicer-dependent manner. More recently, a mutant HEV71 lacking the VSR 3A was shown to be replication-defective and to induce vsiRNA production and RISC-loading in human somatic cells and mice [206]; the partial rescue of the replication defect in Dicer-deficient cells, independent of IFN pathway, was cited as supportive evidence for the functional nature of these vsiRNAs. While these findings imply that the constraints hampering the validation of mammalian antiviral RNAi might be a reflection of the highly potent VSRs effectively blocking the generation of vsiRNAs, which negates any potentiating effect due to Dicer loss or inactivity, many critical questions remain unanswered. Schuster et al. [207] reported that inactivation of the interferon response pathways in differentiated cells by depleting the cytoplasmic sensors like RIG-1 and MDA5 still failed to uncover vsiRNAs and RNAi-mediated antiviral activity.

The first evidence that host cells might employ a miRNA in antiviral defense was provided by Lecellier et al. [208] who showed that human miR-32 restricts the accumulation of the retrovirus primate foamy virus type 1 (PFV-1) in human cells by targeting viral RNA transcripts. Interestingly, the PFV-1 appears to, apparently taking a page from the playbook used by plant viruses that encode VSRs, preempt this host response via the viral protein Tas that globally represses the miRNA biogenesis pathways including the synthesis of miR-32. Thus, an intricate interplay between the cellular miR-32 expression and virus-encoded Tas protein was proposed to determine the success of PFV-1 infection of target cells. Interestingly, miRNAs have also been proposed to function as IFN-inducible antiviral effectors. The human IFNβ was reported to induce the expression of cellular miRNAs miR-196, miR-296, miR-351, miR-431, and miR-448, which displayed seed-sequence complementarity with HCV RNA genome and downregulated virus replication; conversely, the IFNβ was shown to downregulate the HCV cofactor miR-122, thereby causing a reduction in HCV replication [209]. Taking advantage of the availability of Dicer1-deficient mice, Otsuka et al. [210] determined that cellular miRNAs miR-24 and miR-93 display antiviral activity against vesicular stomatitis virus (VSV) by targeting the viral genes encoding large protein (L protein) and phosphoprotein (P protein). The enterovirus EV71 infection induces the expression of miR-296-5p, which targets the coding region of the viral RNA and inhibits viral replication [211]; interestingly, the virus acquired mutations that enabled it to evade miR-296-5p-mediated suppression. More recently, the IFN-induced miR-342-5p was shown to negatively regulate the cholesterol biosynthesis pathway, which is relied upon by several viruses for replication, thus impairing the replication of pathogenic viruses including Influenza A and cytomegalovirus [212].

As in the case of siRNAs, co-evolution with cellular miRNAs in target cells potentially provides the viruses with necessary selection pressure to develop mechanisms to outmaneuver the antiviral activity of these miRNAs. However, despite exposure to multitude of miRNAs in the target cell, viruses have not generally been observed to alter cellular miRNA levels in infected cells. While poxvirus infection coincides with cellular miRNA degradation [213], HIV-1 and influenza A do not appear to antagonize cellular miRNA function [214,215]. Conversely, viruses might simply exploit one defining functional attribute of the miRNAs to evade surveillance. The miRNAs are evolutionarily conserved and, unlike the siRNAs, display partial homology to the target mRNA sequences. However, exact complementarity to the “seed” sequence comprising nucleotides 2–8 of the miRNAs is critical for mRNA target recognition and binding. Hence, even single nucleotide changes acquired by the target mRNAs, including the viral RNAs, may potentially make them immune to any inhibitory action by miRNAs [216]. A striking example for such escape from inhibitory miRNA has been reported in the case of the human T-lymphotropic virus type 1 (HTLV-I), another retrovirus that has recently shown to be controlled by a cellular miRNA. The miR-28-3p was shown to target and prevent the translation of HTLV-1 RNA [217], whereas miR-28-5p, reported to target the HIV-1 RNA, has no binding site on HTLV-1. Interestingly, the ATK-1 strain of HTLV-1 harbors a single-nucleotide polymorphism within the miR-28-3p binding site, and, thus, is resistant to the miR-28-3p. HIV-1 has also been reported to evolve viral quasispecies harboring silent codon changes in order to evade RNAi targeting of the critical viral ORFs [218].

**Figure 2:**
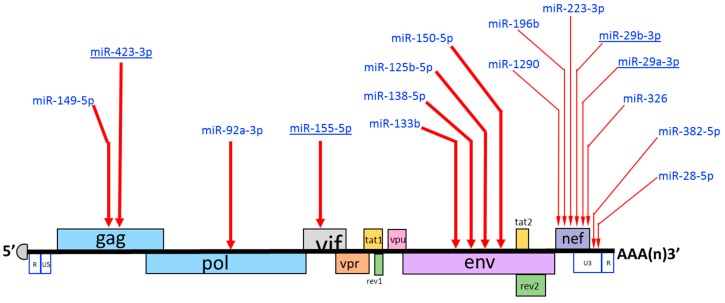
Schematic highlighting the binding sites of cellular miRNAs reported to directly target and bind HIV-1 RNA, and discussed in this review. The cellular miRNAs that are also incorporated into the HIV-1 particles are underlined.

Computational target prediction programs have come to represent one of the favored steps in decoding the function of a miRNA, which largely is defined by that of its target genes. Using a combination of four different miRNA target prediction software, Hariharan et al. [219] identified five sites in the HIV-1 genomic RNA that are potential targets of miRNAs. Interestingly, all these miRNA target sites mapped to the viral RNA regions encoding viral accessory proteins: the *nef* gene harbored target sites for miRNAs miR-29a and miR-29b, the *vpr* for miR-149, the *vif* for miR-324-5p, and *vpu* for miR-378 (Figure 2 and Table 1). In a follow-up study, the authors demonstrated that miR-29a disrupted the expression of viral nef protein and impaired HIV-1 replication in Jurkat cells [220]. Houzet et al. [221], used an in silico approach to identify target sites for 20 cellular miRNAs in HIV-1 RNA, and then experimentally tested the ability of the candidate miRNAs to suppress HIV-1 replication in transfected cells. This led to the narrowing down of the list to five miRNAs: miR-133b, miR-138-5p, miR-326, miR-149-5p, and miR-92a-3p, which all reduced HIV-1 replication by over 40%. Based on experiments that tested the effect of increasing the complementarity between miR-326 and HIV-1 mRNA, the authors contended that the potency of miR-326-mediated repression of HIV-1 replication qualifies for a functionally relevant miRNA–vRNA interaction.

Another widely used strategy to investigate the interplay between miRNAs and HIV-1 has been to analyze changes in cellular miRNA levels in response to HIV-1 infection or downstream host responses. Any such HIV-specific miRNA expression signature may negatively affect HIV-1 replication directly by targeting the virus or indirectly by modulating cellular cofactors or antiviral factors. Yeung et al. [226] employed a high throughput microarray method to quantify the miRNA expression levels in HIV-1-transfected HeLa cells. They reported that, while no cellular miRNAs were upregulated, the HIV-1 infection was accompanied by the downregulation of 312 host miRNAs. RT-PCR analysis of a selective group of four miRNAs from this list—miR-93, miR-148b, miR-221 ad miR-16, supported the microarray data. However, this study did not clarify the mechanistic basis for such extensive downregulation of cellular miRNAs by the virus, i.e., whether all or many of the downregulated cellular miRNAs hold anti-HIV potential. Employing a similar microarray-based approach, Triboulet et al. [181] showed that, while 11 cellular miRNAs (including miR-122, miR-297, miR-370, and miR-373) were upregulated, just two cellular miRNAs, miR-17-5p and miR-20a were downregulated upon HIV-1 infection in the Jurkat cells. Though, neither miR-17-5p nor miR-20a could directly target the viral RNA, both miRNAs had four binding sites located on the 3′-UTR of histone acetyltransferase P300/CBP-associated factor (PCAF), an important cellular cofactor for the viral Tat function. Accordingly, exogenous overexpression of these miRNAs in PBMCs caused substantial depletion of cellular PCAF levels accompanied by impaired HIV-1 replication, whereas blocking the function of the endogenous versions of these miRNAs by antisense inhibitors enhanced PCAF protein expression levels and HIV-1 production. Intriguingly, Whisnant et al. [222] failed to detect any significant alteration in the expression levels of miR-17-5p and miR-20a in HIV-1-infected primary CD4^+^ PBMCs. Though many potential reasons may underlie such opposing observations, this particular discrepancy further supports the notion that findings from studies that utilize monoculture cell lines optimized for in vitro assays may not necessarily reflect the in vivo situation. For instance, Triboulet et al. [181] used the immortalized Jurkat T-cell line and assayed for the miRNA expression levels 21 days post infection by microarray analysis and, at the earliest time point of, seven days post infection by RT-PCR. In contrast, Whisnant et al. (2013) [222] tested the primary CD4^+^ PBMCs 3 days post infection, because, as they reason in their report, the HIV-1-infected T cells in vivo have a half-life of only around 24 h [227]. By comparing the miRNA expression profiles of cells productively or latently infected by HIV-1, Wang et al. [224] identified two cellular miRNAs- miR-196b and miR-1290, that were relatively upregulated in latently infected cells. These two miRNAs were also shown to directly target the 3′UTR of HIV-1 transcripts, while overexpression and knockdown experiments conducted using lab cell lines and CD4^+^ T-cells from HIV-1-infected patients receiving cART revealed the potential of these miRNAs to modulate HIV-1 replication, as evidenced by changes in virus production and infectivity. Based on these results, the authors proposed that miR-196b and miR-1290 may contribute to HIV-1 latency.

A potential link between miRNA expression levels and the recalcitrance of resting memory CD4^+^ T-cells to HIV-1 infection has also been reported. Compared to the activated CD4^+^ T-cells, resting memory CD4^+^ T-cells displayed upregulation of five miRNAs—miR-28, miR-125b, miR-150, miR-223, and miR-382, which negatively targeted the 3′-ends of HIV-1 mRNAs [179]. Accordingly, in resting CD4^+^ T-cells that were infected with HIV-1 infectious clones or were isolated from cART-experienced HIV-1 patients, knockdown of these miRNAs by antisense inhibitors enhanced both viral protein production and virion production. Addtionally, over-expression and knock-down studies showed that miR-125b expression negatively correlated with HIV-1 infection in a T cell line [228]. Besides the miRNAs that appear to modulate HIV-1 infection by directly targeting the viral RNAs, certain miRNAs reportedly play an indirect role by regulating the expression of cellular cofactors in resting CD4^+^ T-cells. The protein levels of the cellular cofactor cyclin T1, which plays critical roles in the viral Tat-mediated transactivation of HIV-1 LTR-driven gene expression, were upregulated independent of the transcript levels upon activation of the resting CD4^+^ T-cells [183]. Interestingly, this upregulation of cyclin T1 was accompanied by a significant downregulation of a group of miRNAs that included miR-27b, miR-29b, miR-150, and miR-223 in activated CD4^+^ T-cells. The authors verified this observation by overexpressing or depleting the miRNAs, which decreased or increased the cyclin T1 protein levels, respectively. However, only the miR-27b was shown to directly regulate the cyclin T1 expression, whereas miR-29b, miR-150, and miR-223 were all proposed to indirectly affect the cyclin T1 levels.

Monocytes are refractory to productive HIV-1 replication, but become progressively susceptible upon differentiation into monocyte-derived macrophages (MDM) or monocyte-derived dendritic cells (MDDC). Though several mechanisms have been proposed to account for this post-entry restriction to HIV-1, as in the case of resting memory CD4^+^ T-cells, a potential links between miRNA expression levels and the recalcitrance of monocytes to HIV-1 infection have been reported. Wang et al. [223] reported that monocytes expressed elevated levels of the cellular miRNAs miR-125b-5p, miR-28-5p, miR-150-5p, miR-223-3p, and miR-382-5p, all previously shown by Huang et al. [179] to impair HIV-1 replication in resting CD4^+^ T-cells. Because knockdown of these miRNAs in monocytes enhanced HIV-1 infection, and miRNA overexpression in MDMs inhibited HIV-1 replication, the authors proposed that the expression levels of these miRNAs determine the susceptibility of monocytes or MDMs to HIV-1.

Interestingly, as in the case of resting CD4^+^ T-cells, cyclin T1 expression was shown to be upregulated upon differentiation of monocytes into MDMs, which was accompanied by robust downregulation of the cellular miR-198 [182]. The miR-198 was shown to directly target the 3′UTR of cyclin T1, and miR-198 overexpression or knockdown in monocytes undergoing differentiation decreased or increased the cyclin T1 protein levels, respectively, with concomitant effects on HIV-1 replication. Expression of another cellular protein called purine-rich element binding protein α (Pur-α), known to promote tat-mediated transactivation, has also been shown to be diminished in the monocytes compared to the MDDCs. This downregulation of Pur-α in monocytes was attributed to the robust expression of a group of miRNAs that included miR-15a, miR-15b, miR-16, miR-20a, miR-106b, and miR-93, which all directly targeted the 3′UTR of Pur-α [229]. While inhibition of these miRNAs in monocytes, via chemically synthesized miRNA inhibitors, caused only a modest enhancement of HIV-1 infection, suppression of endogenous Pur-α expression in MDDCs caused substantial reduction in virus infection. Further, the repression of Pur-α by multiple miRNAs highlights the cooperativity principle that has been proposed to ensure effective target repression [230]. The diminished expression of another cellular cofactor called Vpr(HIV-1)-binding protein (VprBP) in monocytes compared to the MDDCs has also been attributed to the direct targeting of VprBP 3′UTR by the cellular miRNA miR-1236 [231]. Knockdown of miR-1236 in monocytes or overexpression in MDDCs increased or decreased the VprBP protein levels, respectively, with concomitant effects on HIV-1 replication.

The MDMs, though generally susceptible to HIV-1 infection, are known to become relatively recalcitrant to HIV-1 upon stimulation with the Toll-like receptor 4 (TLR4) agonist polyinosine-polycytidylic acid (poly(I:C)) or Toll-like receptor 4 (TLR4) agonist lipopolysaccharide (LPC). Swaminathan et al. [232] demonstrated that this TLR3/4 stimulation-induced suppression of HIV-1 infection in MDMs could be attributed to enhanced expression of the cellular miR-155, which interfered with the post-entry and pre-integration events in HIV-1 replication, as evidenced from accumulation of late RT products and diminished proviral integration and 2-LTR circles. Interestingly, poly(I:C) stimulation or miR-155 overexpression led to repression of several HDFs implicated in trafficking and nuclear import of PICs, including ADAM10, TNPO3, Nup153, and LEDGF/p75, all of which were shown to be directly targeted at their 3′UTR by the miR-155. The cellular miR-155 has also been shown to reinforce HIV-1 latency by targeting the 3′UTR of the E3 ubiquitin ligase TRIM32 transcript and repressing its expression, thereby blocking its promotion of reactivation of latent HIV-1 [233]. The recent report by Lodge et al. [234] adds two cellular miRNAs, miR-221 and miR-22, to the repertoire of miRNAs acting as effectors of antiviral host response in MDMs. The miR-221 and miR-22 were both upregulated in MDMs, and they were shown to target the 3′UTR of CD4 transcript and suppress both the mRNA and protein levels, thereby impairing HIV-1 entry into MDMs.

Astrocyte is another cell type reported to be non-productively infected by HIV-1, in a CD4-independent manner. Recently, elevated levels of the anti-HIV-1 restriction factor sterile alpha motif and histidine/aspartic acid domain-containing protein 1 (SAMHD1) was reported to contribute to the restriction of virus replication in cultured astrocytes [235]. An earlier report by Jin et al. [236] had demonstrated the downregulation of SAMHD1 levels by miR-181a in a monocytic cell line (THP-1) and T-cell line (Jurkat). Interestingly, the SAMHD1 mRNA was shown to harbor binding sites for two cellular miRNAs—miR-181a and miR-155, both of which were poorly expressed in astrocytes compared to the microglial cells [235]. Accordingly, exogenous overexpression of these two miRNAs led to reduction in SAMHD1 levels and, consequently, enhanced HIV-1 replication in vitro. Intriguingly, and in a notable demonstration of a potential disconnect between miRNA-mediated effects observed in vitro and in vivo, Witwer et al. [237] reported a lack of correlation between the in vivo levels of SAMHD1 and miR-181a or miR-155 in a well-characterized SIV model of HIV-1.

Nathans et al. [180] pursued an integrative approach wherein they performed miRNA target prediction analysis, which revealed that the HIV-1 3′-UTR harbored target binding sites for at least 11 cellular miRNAs, and then followed it up by testing the data by miRNA microarray analysis, which showed that the miR-29a was highly expressed in HIV-infected T-cells. The miR-29a target site was highly conserved in various HIV-1 subtypes and was shown to directly target and prevent the expression of the viral mRNA. Investigations on underlying mechanistic basis revealed that miR-29a promotes the sequestration or association of the viral mRNAs with P-bodies and RISC proteins. Interestingly, physical and functional interactions between the HIV-1 mRNA and the RISC complex effector proteins have been reported by Chable-Bessia et al. [238]. The authors showed that some components of the RISC complex, including GW182, associate with HIV-1 mRNA and block its association with polysomes, thereby impairing viral gene expression. Attempts have also been made to dissect HIV-1 interaction with host miRNAs in a wider array of clinically relevant target cells. Sun et al. [225] performed a comprehensive analysis of any perturbations to the host miRNA expression levels during acute HIV-1 infection of a variety of target cells including CD4^+^CD8^−^ PBMCs, CEM, and Jurkat. The authors neither observed any HIV-induced global miRNA deregulation nor could they recapitulate the findings from some miRNA profiling studies reported earlier [181,226]. Interestingly, while these earlier studies had used small RNA-enriched samples for miRNA profiling analysis, Sun et al. [225] relied on total RNA samples instead. Even though downregulation of miR-29 family members were observed [180,220], the authors contended that any suppressive effect by the miRNA is attenuated by the secondary structure of the viral LTR target sequence. A recent report by Adoro et al. [239] provides an alternative conceptual framework to decode the anti-HIV activity of miR-29. In experiments targeted towards deciphering the mechanistic details underlying the IL-21-mediated inhibition of HIV-1 replication, the authors showed that IL-21 promotes miR-29 biogenesis in a STAT3-dependent fashion and reverses the HIV-induced downregulation of miR-29.

Intriguingly, besides the cellular miRNAs, the components of the cellular machinery involved in the biogenesis and execution of the miRNA-mediated gene silencing have also been shown to suppress HIV-1 replication [240]. In a key study, Triboulet et al. [181] reported that knockdown of the endogenous Dicer resulted in enhanced HIV-1 replication and virus production. Nathans et al. [180] reported that depleting Dicer or Drosha in the producer cell enhanced HIV-1 production, whereas depleting either of those two enzymes in the target cell increased viral infectivity. However, in a recent study, Bogerd et al. [199] demonstrated that the replication of many viruses including HIV-1 was not enhanced in human cell lines that lack a functional Dicer enzyme and therefore deficient in the generation of miRNAs and siRNAs; these findings were recently corroborated by Eckenfelder et al. [241]. Direct interaction of miRISC protein components with the HIV-1 RNAs, in a miRNA-independent manner, has also been reported [180,238,241,242]. In HIV-1-infected CD4^+^ T-cells, the AGO2 protein was shown to directly and preferentially bind, in a miRNA- and Dicer-independent manner, near splice donor sites in the unspliced viral mRNA [241]. This AGO2-viral mRNA interaction impaired the production of spliced HIV-1 mRNAs, thereby implicating the AGO2 protein in HIV-1 mRNA splicing events. Interestingly, Vongrad et al. [243] reported an absence of such interaction between the HIV-1 RNA and AGO2 in monocyte-derived macrophages (MDM), suggesting that HIV-1 is not targeted by the canonical RNAi pathway in MDMs.

Inhibition of HIV-1 replication by cellular miRNAs via non-canonical (i.e., gene silencing-independent) pathways has been reported. Overexpression of miR-146a or miR-888—two exogenous miRNAs neither present in the 293T cells nor possess any known target sites on HIV-1 mRNAs—impaired HIV-1 production but did not alter intracellular Gag protein expression, thereby implicating an RNAi-independent mechanism [244]. Super resolution microscopy analyses revealed that the overexpressed miRNA competes with viral RNA for Gag binding, and the aberrantly assembled RNA-protein complexes at the PM were endocytosed in a dynamin-dependent manner into large intracellular vacuoles. Interestingly, coexpressing target mRNAs of these exogenous miRNAs relieved the inhibitory effect on Gag assembly and virus production. Further, knockdown of AGO2 induced a phenotype comparable to that caused by the exogenous miRNAs. These observations suggest that endogenous miRNAs when unbound to miRISC might bind Gag disrupting its assembly and virus production. Importantly, knockdown of Dicer or Drosha reduced the numbers of Gag-enriched intracellular compartments and promoted robust virus particle production. Taken together, these findings provide a potential mechanistic framework for explaining the inhibitory effects exerted by endogenous Dicer and Drosha on HIV-1 replication. Importantly, these findings also provide a cautionary note on the physiological relevance and interpretation of data obtained from miRNA overexpression experiments to test potential antiviral effect, i.e., any gene silencing-independent effects of candidate cellular miRNAs must be accounted for before conferring functional relevance to miRNA-mediated antiviral activity.

## 7. Does HIV-1 Encode miRNAs?

Like their hosts, increasing numbers of viruses are being reported to encode miRNAs. Majority of the viral miRNAs discovered so far are encoded by DNA viruses that replicate and assemble in the host cell nucleus, including Herpesviruses, polyomaviruses, and adenoviruses (miRBase, Release 21). Notably, over 90% of these miRNAs are conserved between related species and are encoded by Herpesviridae family that include Epstein–Barr virus (EBV), Kaposi’s sarcoma-associated herpesvirus (KSHV), Herpes simplex virus (HSV), and Human cytomegalovirus (HCMV). These viral miRNAs have been shown to regulate viral and host gene expression that, by modulating metabolic processes and/or the host immune response, generally promote viral replication and, in certain cases, viral latency.

The evidence that RNA viruses encode miRNAs is highly controversial [222,245,246,247,248,249]. The reservations against the feasibility of RNA viruses encoding miRNAs mostly invoke the distinct subcellular locations of the virus replication cycle and miRNA biogenesis pathway. In addition, potential cleavage of the viral genomic RNA in cis by the miRNA processing machinery poses a tangible bottleneck limiting productive virus replication. For example, the first step of the canonical miRNA biogenesis occurs in nucleus, wherein Drosha-mediated cleavage of pri-miRNA generates the pre-miRNA. Because majority of RNA viruses complete their replication in the cytoplasm, this potentially precludes them from accessing the nuclear-localized miRNA biogenesis machinery to generate their own miRNAs. Additionally, the endonucleolytic cleavage of precursor transcripts during miRNA-generating pathways might adversely target the viral genomic RNA or mRNAs. Nevertheless, to date, three retroviruses—bovine leukemia virus (BLV), simian foamy virus of the African green monkey (SFVagm), and the bovine foamy virus (BFV)—have been shown to circumvent the canonical nuclear Drosha-mediated RNA processing step. These viruses harbor Pol III promoters in their genome and accordingly employ a non-canonical strategy wherein RNA polymerase III, instead of RNA Pol II, are usurped to transcribe viral sub-genomic transcripts harboring pre-miRNA hairpins, which are then processed into mature miRNAs in Drosha-independent manner [250,251,252,253,254]. It should be noted that these studies were preceded by an important report showing that the murine herpesvirus 68 encodes several miRNAs that are products of transcription by Pol III [255]. The BLV’s reliance on Pol III to express miRNAs has been proposed to maintain its Pol II-transcribed coding genes silent and thereby evade the cellular immune surveillance. While BLV replication proceeds despite weak viral transcription levels [256,257], robustly generated BLV miRNAs reportedly constitute up to 40% of total cellular miRNAs [251]. Further, at least one of the BLV-encoded miRNAs appears to share common traits with miRNAs encoded by some DNA viruses. Comparable to the KSHV- and MDV-encoded miRNAs that function as viral analogs of the cellular oncomir miR-155, the BLV-encoded miR-B4 and the cellular oncomir miR-29a share an identical seed sequence, and both contribute to oncogenesis by targeting the same cellular mRNAs. The SFVagm reportedly encodes six miRNAs transcribed from its LTR region by Pol III. The seed sequences of two of these miRNAs, SFVagm-miR-S4-3p and SFVagm-miR-S6-3p, are homologous to the cellular miR-155 and innate immunity suppressor miR-132, respectively. In the case of BFV, it employs an unusual strategy wherein Pol III transcribes a single ~122-nucleotides-long pri-miRNA, which is then cleaved into two pre-miRNAs that are further processed to yield three different mature miRNAs. The first retroviral miRNA identified as one generated via the canonical miRNA biogenesis pathway (using Pol II) was transcribed from the viral E-element/exogenous virus-specific region (XSR) of the retrovirus avian leucosis virus subgroup J (ALV-J) [258]. However, the biological function of this E/XSR-miRNA in the ALV-J-associated myeloid leukosis is yet to be deciphered. It also remains to be seen if retroviruses encode miRNA to modulate the host machinery for productive replication and transmission.

The notion that HIV-1 may encode miRNAs stemmed from the recognition that the viral genome harbored secondary structures including stem-loops that mirror the substrates of the cellular miRNA biogenesis machinery. Earliest investigations along that line on HIV-encoded candidate miRNAs relied on computational approaches. Bennasser et al. [259] reported that stem-loop structures located in five regions of the HIV-1 genomic RNA—trans-activation response (TAR) element, Gag-CA, Gag-Pol frameshift, Nef, and 3′-LTR—are potential miRNA-yielding hotspots encoding up to 10 miRNAs. Couturier and Root-Bernstein [260] probed for sequence complementarity between the proviral genome and the mRNA of target cells, which led to the prediction that HIV-1 might encode miRNAs capable of blocking the production of CD4, CD28 and certain interleukins. Omoto et al. [261] reported that HIV-1 encodes miR-N367 from a 70-nucleotide-long structure in the Nef/LTR overlapping region. The miR-N367 was subsequently shown to target the negative response element of the LTR U3 region and inhibit the LTR’s promoter activity [262]. However, miR-N367 was not picked up in screens for HIV-encoded miRNAs in infected HeLa cells [255] and in chronically-infected T-cells [245]. Another reportedly HIV-encoded miRNA miR-H1 is derived from an 81-nucleotide stem-loop structure located downstream of the two NF-kB-binding sites in the viral LTR, and was shown to down-regulate the expression of the apoptosis antagonizing transcription factor (*AATF*) by cleaving its 12th exon [263]. Intriguingly, Lamers et al. [264] reported substantial sequence variability of both the pre-miRNA and mature miR-H1 in over 1000 HIV-1 sequences derived from post-mortem tissue samples of several HIV-1 patients. The Dicer enzyme was reported to bind and cleave the HIV-1 TAR stem-loop structure, and the resulting ~22-nucleotides-long small RNA species, termed TAR-derived miRNA were proposed to promote viral latency by chromatin remodeling of the LTR [265] and to preclude death of HIV-infected cells by downregulating the proapoptotic cellular genes excision repair cross complementation group 1 (*ERCC1*) and immediate early response 3 (*IER3*) [266]. Ouellet et al. [267] reported that asymmetrical processing of the HIV-1 TAR element by Dicer yielded two additional miRNAs, namely miR-TAR-5p and miR-TAR-3p, in HIV-infected cell lines and primary CD4^+^ T-cells. In a follow-up study, the TAR miRNAs were shown to target host genes including caspase 8 (*CASP8*), Ikaros family Zinc Finger 3 (*Aiolos*), Ikaros family Zinc Finger 1 (*IKZF1*), and nucleophosmin 1 (NPM), thereby preventing apoptosis of HIV-infected cells and promoting cell survival and viral replication [268]. Interestingly, exosomes from HIV-infected cells and patient sera harbored TAR RNAs (precursor of TAR miRNAs), and these exosomes down-regulated BCL2 Like 11 (Bim) and CDK9 protein levels and thereby apoptosis in recipient cells [269]. The RT region of the HIV-1 genome was shown by Zhang et al. [270] to encode a miRNA named miR-H3 that targets the TATA box in the viral 5′-LTR promoter. While miR-H3 expression upregulated viral RNA transcription and protein production, mutations in the miR-H3 encoding region impaired HIV-1 replication.

Next-generation sequencing technologies are increasingly being used to validate the reported HIV-1 miRNAs and to address the contentious question of whether HIV-1 is capable of encoding miRNAs. Schopman et al. [271] used sequencing by oligonucleotide ligation and detection (SOLiD) technology-based deep sequencing approach to screen for small RNAs in HIV-infected SupT1 cells. Approximately one percent of the total small RNAs identified mapped to the HIV-1 RNA genome. A majority of these ~18–21-nucleotides-long candidate viral-encoded miRNAs aligned with the viral positive sense RNA, while a small segment corresponded to the viral negative sense RNA. In contrast, a deep sequencing-based analysis using two different HIV-infected cell lines (TZM-bl and CD8166) and two types of primary human cells (CD4^+^ PBMCs and macrophages) did not uncover any HIV-encoded miRNAs [253]. These authors contend that the candidate HIV-specific small RNAs fail to satisfy three criteria for authentic viral miRNAs. First, all other viral miRNAs reported to date are generally derived from fewer locations in their respective genome. However, the HIV-specific small RNAs recovered by Whisnant et al. [253] correspond to the entire proviral genome. Further, unlike in the case of other viral miRNAs, only a minor population (<~1%) of the small RNAs in HIV-infected cells aligned with the viral genome. Second, miRNAs typically range in size from ~20–24 nucleotides in length. However, the small RNAs that align with the HIV-1 genome exhibited a random size distribution with a majority <~20 nucleotides in length. Third, the authors contend that the TAR stem, source of several miRNAs reported, lacks several key defining characteristics of canonical pri-miRNA stem loops. The TAR stem is only ~24 bp long, the terminal loop is just six nucleotides, and it is located at the 5′-end of viral mRNAs. In contrast, the canonical pri-miRNA stem is ~33 bp long, the terminal loop is over 10 nucleotides, and both the 5′- and 3′-ends of the stem is flanked by unstructured RNA sequences. Further, comparing cellular miRNA expression profiles of HIV-infected CD4^+^ PBMCs 72 h post infection, a time point well beyond the reported half-life (~24 h) of virus-infected T-cells in vivo, with that of uninfected control cells revealed no difference (greater than two-fold). To determine whether HIV-1 has successfully evolved out potentially detrimental miRNA-binding sites on its genome, photoactivatable ribonucleoside-enhanced cross-linking and immunoprecipitation (PAR-CLIP) technique was used to determine miRNA-directed RISC-binding sites on viral genome in infected non-target cell types. Unexpectedly, despite making up 10–50% of the total mRNA population in these infected cells, only ~0.3% of the viral transcripts harbored RISC-binding sites. Among these, only a few cellular miRNAs including miR-423, miR-301a, and miR-155 bound viral transcripts and reduced their expression. The authors proposed that evolutionary constraints posed by cellular miRNAs with antiviral properties likely forced the viral RNA to adopt extensive secondary structures that mask miRNA-binding sites. Considering all their findings, the authors concluded that HIV-1 neither encodes miRNAs nor strongly influences cellular miRNA expression, at least early after infection. Vongrad et al. [243] came to similar conclusions when they failed to detect incorporation of HIV-1-derived sncRNAs or any viral target sequences into the AGO2-RISC complex.

## 8. Concluding Remarks and Outstanding Questions

Extraordinary progress in deciphering the biology of cellular miRNAs, since their discovery 25 years back [272], offers a great opportunity to understand the role of these sncRNAs in virus-host interactions. Accumulating evidence suggest that cellular miRNAs play important roles in viral life cycle by promoting or inhibiting virus replication. Accordingly, cellular miRNAs that can directly inhibit HIV-1 replication by targeting the vRNA and/or viral mRNAs, and cellular miRNAs that can indirectly affect HIV-1 infection by targeting HDFs have been reported (Table 2). However, the efficacy and the physiological relevance of any such endogenous cellular miRNA-mediated effects on HIV-1 replication, and the ability of HIV-1 genome to encode miRNAs remain contentious, albeit enriching and important, areas of ongoing research [273]. Several outstanding questions need special attention to better understand the role of miRNAs in HIV-1 infection.

**Figure 3:**
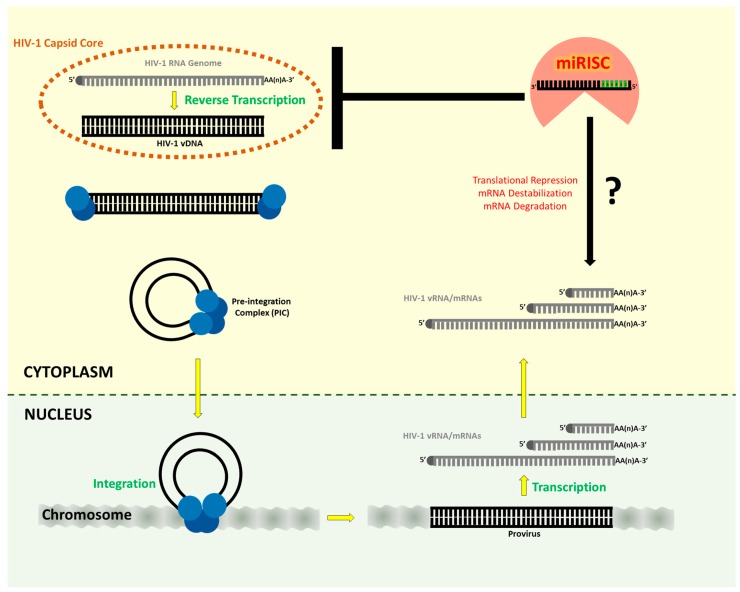
Schematic showing different stages of HIV-1 replication in a target cell and the potential site(s) of miRISC action. While the incoming HIV-1 RNA genome is protected by the viral capsid shell, thus precluding any access to the miRISC, the viral transcripts exported from the nucleus into the cytoplasm are potentially accessible and hence are viable targets of miRISC. However, it remains unclear whether the endogenous levels of cellular miRNAs are sufficient to effectively counter the high number of viral transcripts typically produced during HIV-1 infection. **Yellow arrow** = Indicates/depicts progression of the different stages of HIV-1 replication. **Black arrow** = Indicates/depicts the potential interaction of miRNAs with the HIV-1 RNAs. **Black T bar** = Indicates/depicts the protection provided by the viral capsid shell to the viral RNA genome against the cellular miRNAs.

Are the copy numbers of cellular anti-HIV miRNAs in target cells sufficient to exert a consequential effect on the HIV-1 replication? At least 100 copies of miRNA is reportedly required to effectively downregulate a cellular target RNA [274,275]. This ratio is likely to be far greater when the target is vRNA or viral mRNA. A single HIV-infected target cell is estimated to produce over 10,000 progeny virus particles within one to two days [276], which would require the generation of at least 20,000 vRNA molecules for encapsidation purpose alone, not taking into account the additional number of viral mRNAs necessary for the synthesis of viral proteins (Figure 3). Indeed, analysis by Whisnant et al. [222] revealed the presence of around 20,000 viral transcripts per HIV-1-infected T-cell. Further, miRISC binding to HIV-1 transcripts were reported to be 100-fold less efficient than miRNA binding to cellular mRNAs in HIV-1-infected cells [222]. Interestingly, engineering of artificial miRNA target sites into the HIV-1 RNA led to up to 40-fold increase in the packaging of the cognate miRNAs into the virus particle and inhibited virion infectivity [277]. Further, miRISC binding to HIV-1 transcripts were reported to be 100-fold less efficient than miRISC binding to cellular mRNAs in HIV-1-infected cells [222]. Interestingly, engineering of artificial miRNA target sites into the HIV-1 RNA led to ~40-fold increase in the packaging of the cognate miRNAs into the virus particle and inhibited virion infectivity [276]. Compared to the investigations on determining whether HIV-1 infection alters cellular miRNA signatures, information on the levels of the cellular miRNAs reported to alter HIV-1 replication is very limited. A potential contributing factor is the prevalent practice of reporting miRNA levels from qPCR-based assays as fold-change. Instead, assessing and reporting miRNA induction based on miRNA copy numbers might preclude inadvertent implication of physiologically and/or pathologically irrelevant miRNAs in HIV-1 infection. Further, due to partial complementarity of the miRNA: mRNA interaction, a cellular miRNA typically downregulates the production of a target protein by less than two-fold [278,279]. Hence, even if the expression level of an endogenous candidate anti-HIV miRNA reaches such threshold, and not considering the turnover rate of that miRNA [280], it remains unclear whether the projected 50% reduction in HIV-1 gene expression could negatively impact the virus replication and/or pathogenesis in vivo, in any consequential manner. Expression of endogenous anti-HIV miRNAs sufficient to achieve a complete repression of the viral RNAs has generally not been demonstrated in infected target cells. Also, the kinetics of the miRNA-mediated activity appears not optimal for acute response to virus infection [281].

Do HIV-1 RNAs/mRNAs function as molecular sponges of human miRNAs, thereby relieving translational repression of specific cellular cofactors? Virus replication in target cell produces vRNAs and viral mRNAs for encapsidation and/or generation of viral proteins. The “miRNA sponges” are exogenously-expressed artificial RNA transcripts harboring binding sites for specific target miRNAs, and the resulting competitive inhibition (sponging off) of the miRNA can lead to the derepression of its target cellular mRNAs [282,283]. The occurrence of endogenous miRNA sponges (competitive endogenous RNA, ceRNA) and their ability to effectively derepress the target mRNAs in vivo is an equally exciting and contentious area of ongoing research [284,285,286]. However, this has led to the proposal that viral RNAs, harboring shared miRNA binding sites with cellular mRNAs, can compete and sequester the cognate cellular miRNAs, thereby selectively altering the stability and translational efficiency of the target cellular mRNAs in the infected cells. It has been speculated that viruses can employ such “miRNA sponge” effect to counter cellular miRNAs towards establishing a productive infection and promote viral pathogenesis. Indeed, the *Herpesvirus saimiri* (HSV)-derived non-coding transcripts called *H. saimiri* U-rich RNAs (HSURs) were reported to interact with and downregulate the cellular miR-27, thereby altering the expression of its target genes [287]. Similar virus-directed miRNA sponge effects have been reported to occur in infections by other viruses including HBV [172,288], murine cytomegalovirus (MCMV) [289,290], HCMV [291], HCV [170], and human papillomaviruses (HPV). However, HIV-1 has not yet been demonstrated to employ its transcripts as miRNA sponges to modulate the expression of host genes.

Does HIV-1 adapt and evolve resistance to anti-viral miRNAs? Drug-resistant mutant HIV-1 strains continue to pose a significant challenge to the cART and management of HIV/AIDS. So, one would presume that HIV-1 would eventually acquire mutations in its vRNA genome to evade the sequence-specific repression by anti-HIV cellular miRNAs. RNAi-mediated degradation of vRNA genome has been employed to inhibit replication of many viruses including HIV-1; specifically, the tat, rev and gag coding regions of HIV-1 RNA have been targeted by RNAi to inhibit virus replication [292,293,294,295,296,297,298,299,300,301,302,303,304]. Despite the RNAi exhibiting potent antiviral activity towards HIV-1, the virus has been shown to eventually escape the RNAi-mediated repression by acquiring mutations within the siRNA-target sequence [297,299,300,304]. Indeed, HIV-1 has been shown even to counter combinatorial RNAi approaches by acquiring mutations in multiple siRNA-target sites [304,305].

Is undue reliance on miRNA target prediction programs contributing to reporting of miRNA-mRNA interactions that are probably physiologically irrelevant in the context of HIV-1 replication? Existing computational methods used to predict miRNA targets generally rely on sequence similarity, including perfect Watson–Crick base pairing between the seed sequence of the miRNA and the target mRNA [107]. However, the occurrence of a complementary seed sequence in the target mRNA does not by default translate into effective miRNA binding [143,306]. Accordingly, the false positive rates of the popular prediction algorithms range between 20–50% [278,307]. Conversely, the inability of the miRNA target prediction tools to recognize non-canonical interactions between miRNAs and their mRNA targets [308] contribute to equally non-productive false negatives. Furthermore, the local 3D structure on the target mRNA, especially in the case of highly structured HIV-1 RNA [53], can significantly impact the accessibility of the miRNA-binding site to the miRISC and thus the outcome of the miRNA-mRNA interaction [144,309,310]. In other words, even the best available prediction algorithms may not be modeling what really happens inside an HIV-infected target cell.

Collectively, information on any functional interplay between HIV-1 and cellular miRNA pathways provides a significant and timely opportunity to reveal new insights into virus–host interactions governing the virus replication and viral pathogenesis. The potential discoveries hold great promise for developing novel anti-HIV-1 therapies [46]. However, such promises can only be materialized by systematically addressing the key knowledge gaps highlighted above.

## Figures and Tables

**Table 1:** Details of cellular miRNAs reported to target HIV-1 RNA, and discussed in this review.

	miRNA	Sequence (5′-3′)	Target Region on HIV-1 RNA	Targeted Sequence on HIV-1 RNA (5′-3′)	Effect on HIV-1 Replication	Reference
1	miR-149-5p	UCUGGCUCCGUGUCUUCACUCCC	Gag	GGGAGTGGGGGGACCCGGCCATA	Negative	Houzet et al. 2012 [221]
2	miR-423-3p	AGCUCGGUCUGAGGCCCCUCAGU	Gag	UAUAAAACUCUAAGAGCCGAGCA	Negative	Whisnant et al. 2013 [222]
3	miR-92a-3p	UAUUGCACUUGUCCCGGCCUGU	Pol	GCATTGGGAATCATTCAAGCACAACCA	Negative	Houzet et al. 2012 [221]
4	miR-155-5p	UUAAUGCUAAUCGUGAUAGGGGU	Vif	GUACUUGGCACUAGCAGCAUUAA	Negative	Whisnant et al. 2013 [222]
5	miR-133b	UUUGGUCCCCUUCAACCAGCUA	Env	GACCTGGATGGAGTGGGACAGA	Negative	Houzet et al. 2012 [221]
6	miR-138-5p	AGCUGGUGUUGUGAAUCAGGCCG	Env	ATTGAAGAATCGCAAAACCAGCA	Negative	Houzet et al. 2012 [221]
7	miR-125b-5p	UCCCUGAGACCCUAACUUGUGA	Env	ACGAGGAUUGUGGAACUUCUGGGACGCAGGGG	Negative	Huang et al. 2007 [179]
8	miR-150-5p	UCUCCCAACCCUUGUACCAGUG	Env	UCUGGGACGCAGGGGGUGGGAA	Negative	Huang et al. 2007 [179]; Wang et al. 2009 [223]
9	miR-1290	UGGAUUUUUGGAUCAGGGA	Nef/3′UTR	UCCUUGAUCTGUGGAUCUA	Negative	Wang et al. 2015 [224]
10	miR-196b	UAGGUAGUUUCCUGUUGUUGGG	Nef/3′UTR	CUACCACACACAAGGCUACUUC	Negative	Wang et al. 2015 [224]
11	miR-223-3p	UGUCAGUUUGUCAAAUACCCCA	Nef/3′UTR	AGGGGUCAGAUAUCCACUGACC	Negative	Huang et al. 2007 [179]; Sun et al. 2012 [225]; Wang et al. 2009 [223]
12	miR-29b-3p	UAGCACCAUUUGAAAUCAGUGUU	Nef/3′UTR	AUACCCACUGACCUUUGGAUGGUGCUU	Negative	Sun et al. 2012 [225]
13	miR-29a-3p	UAGCACCAUCUGAAAUCGGUUA	Nef/3′UTR	CACUGACCUUUGGAUGGUGCUU	Negative	Ahluwalia et al. 2008 [220]; Nathans et al. 2009 [180]
14	miR-326	CCUCUGGGCCCUUCCUCCAG	Nef/3′UTR	TGGGATGGAGGACCCGGAGG	Negative	Houzet et al. 2012 [221]
15	miR-382-5p	GAAGUUGUUCGUGGUGGAUUCG	U3/3′UTR	CGAGCUUGCUACAAGGGACUUU	Negative	Huang et al. 2007 [179]; Wang et al. 2009 [223]
16	miR-28-5p	AAGGAGCUCACAGUCUAUUGAG	U3/3′UTR	AUCUGAGCCUGGGAGCUCUC	Negative	Huang et al. 2007 [179]; Wang et al. 2009 [223]

**Table 2:** Cellular miRNAs reported to target the host-dependency factors (HDFs) and modulate HIV-1 replication negatively or positively, and discussed in this review.

	miRNA	Target HDF/Cellular mRNA	Effect on HIV-1 Replication	Reference
1	miR-17-5p	PCAF 3′UTR	Reduction in HIV-1 infection in PBMCs and Jurkat cells	Triboulet et al. 2007 [181]
2	miR-20a	PCAF 3′UTR	Reduction in HIV-1 infection in PBMCs and Jurkat cells	Triboulet et al. 2007 [181]
3	miR-198	Cyclin T1 3′UTR	Impaired HIV-1 replication in monocytes	Sung and Rice, 2009 [182]
4	miR-15a	Pur-alpha 3′UTR	Impaired HIV-1 replication in monocytes	Shen et al. 2012 [229]
5	miR-15b	Pur-alpha 3′UTR	Impaired HIV-1 replication in monocytes	Shen et al. 2012 [229]
6	miR-16	Pur-alpha 3′UTR	Impaired HIV-1 replication in monocytes	Shen et al. 2012 [229]
7	miR-20a	Pur-alpha 3′UTR	Impaired HIV-1 replication in monocytes	Shen et al. 2012 [229]
8	miR-93	Pur-alpha 3′UTR	Impaired HIV-1 replication in monocytes	Shen et al. 2012 [229]
9	miR-106b	Pur-alpha 3′UTR	Impaired HIV-1 replication in monocytes	Shen et al. 2012 [229]
10	miR-155	ADAM10 3′UTR, NUP153 3′UTR, LEDGF/p75 3′UTRTRIM32 3′UTR SAMHD1	Reduction in HIV-1 late RT products and viral DNA integration in MDMs Promotes reactivation of latent HIV-1 via NF-kB signaling in J-Lat 5A8 cells Overexpression of miR-155 enhanced HIV-1 replication in astrocytes	Swaminathan et al. 2012 [232] Ruelas et al. 2015 [233] Pilakka-Kanthikeel et al. 2015 [235]
11	miR-27b	Cyclin T1 3′UTR	Impaired HIV-1 replication in resting CD4+ T-cells	Chiang et al. 2012 [183]
12	miR-29b	Cyclin T1 3′UTR	Impaired HIV-1 replication in resting CD4+ T-cells	Chiang et al. 2012 [183]
13	miR-150	Cyclin T1 3′UTR	Impaired HIV-1 replication in resting CD4+ T-cells	Chiang et al. 2012 [183]
14	miR-223	Cyclin T1 3′UTR	Impaired HIV-1 replication in resting CD4+ T-cells	Chiang et al. 2012 [183]
15	miR-1236	VprBP 3′UTR	Impaired HIV-1 replication in monocytes	Ma et al. 2014 [231]
16	miR-181a	SAMHD1	Overexpression of miR-181a enhanced HIV-1 replication in astrocytes	Pilakka-Kanthikeel et al. 2015 [235]
17	miR-132	MeCP2	Increased HIV-1 infection in Jurkat T-cells	Chiang et al. 2013 [178]
18	Let-7c	P21 3′UTR	Increased HIV-1 replication in JLTRG-R5 and HeLa-CCR5 cells	Farberov et al. 2015 [190]
19	miR-124a	TASK1 3′UTR	Increased HIV-1 replication in JLTRG-R5 and HeLa-CCR5 cells	Farberov et al. 2015 [190]
20	miR-34a-5p	TASK1 3′UTR	Increased HIV-1 replication in JLTRG-R5 and HeLa-CCR5 cells	Farberov et al. 2015 [190]
21	miR-34c-5p	Several genes involved in TCR signaling and activation of naïve CD4+ T-cells	Increased HIV-1 replication in Jurkat T-cells	Amaral et al. 2017 [191]
22	miR-221	CD4 3′UTR	Inhibition of HIV-1 entry in macrophages	Lodge et al. 2017 [234]
23	miR-222	CD4 3′UTR	Inhibition of HIV-1 entry in macrophages	Lodge et al. 2017 [234]
